# Pendulum test in chronic hemiplegic stroke population: additional ambulatory information beyond spasticity

**DOI:** 10.1038/s41598-021-94108-5

**Published:** 2021-07-20

**Authors:** Yin-Kai Dean Huang, Wei Li, Yi-Lin Chou, Erica Shih-Wei Hung, Jiunn-Horng Kang

**Affiliations:** 1 Department of Physical Medicine and Rehabilitation, Taipei Medical University Hospital, Taipei, Taiwan; 2 Department of Physical Medicine and Rehabilitation, School of Medicine, College of Medicine, Taipei Medical University, Taipei, Taiwan; 3 Professional Master Program in Artificial Intelligence in Medicine, College of Medicine, Taipei Medical University, Taipei, Taiwan

**Keywords:** Stroke, Stroke, Disability, Physical examination

## Abstract

Spasticity measured by manual tests, such as modified Ashworth scale (MAS), may not sufficiently reflect mobility function in stroke survivors. This study aims to identify additional ambulatory information provided by the pendulum test. Clinical assessments including Brünnstrom recovery stage, manual muscle test, MAS, Tinetti test (TT), Timed up and go test, 10-m walk test (10-MWT), and Barthel index were applied to 40 ambulant chronic stroke patients. The pendular parameters, first swing excursion (FSE) and relaxation index (RI), were extracted by an electrogoniometer. The correlations among these variables were analyzed by the Spearman and Pearson partial correlation tests. After controlling the factor of motor recovery (Brünnstrom recovery stage), the MAS of paretic knee extensor was negatively correlated with the gait score of TT (r =  − 0.355, *p* = 0.027), while the FSE revealed positive correlations to the balance score of TT (r = 0.378, *p* = 0.018). RI were associated with the comfortable speed of 10-MWT (r = 0.367, *p* = 0.022). These results suggest a decrease of knee extensor spasticity links to a better gait and balance in chronic stroke patients. The pendular parameters can provide additional ambulatory information, as complementary to the MAS. The pendulum test can be a potential tool for patient selection and outcome assessment after spasticity treatments in chronic stroke population.

## Introduction

Spasticity is a common problem following a stroke and may affect walking ability. It is manifested by a “catch and release” phenomenon during rapid stretching a muscle^1^. This velocity-dependent nature is noticeable in 19–28% of patients within 3 months of stroke, and 34% at 18 months^2^. Spasticity may impede recovery of balance and gait^3^, escalate risk of fall^4^, and reduce quality of life^5^. Attention on spasticity is because functional improvement can be achieved with treatments, such as neurotomy^6^ or botulinum toxin injection^7^. However, some studies concluded spasticity did not contribute to walking disability after stroke^8–10^. The influence of spasticity on mobility dysfunction may be various by muscle groups, postures, or circumstances (static or dynamic). Appropriate measurement is critical for effective management.

Knee spasticity had been linked to balance and gait performance in stroke survivors. Abnormal knee posture was reported correlated with increased spasticity (both knee flexor and extensor), weak knee flexor, and poor standing balance^11^. Overactivity of knee extensor disabled knee flexion during the swing phase of gait, resulting in gait abnormality, which was not predictable by static spasticity^12^. Assessment of dynamic spasticity could be essential for patient selection. For example, Botulinum toxin injection to rectus femoris increased knee flexion and improved locomotion function in stroke patients with stiff knees^13–16^.

Assessment of dynamic knee spasticity is challenging in clinical setting. Modified Ashworth Scale (MAS) is a widely used manual test to grade muscle tone abnormality on a 0–4 (with grade 1+)^17^ or 0–5 scale^18^ by a “stretch-reflex” test. However, the spasticity provoked by stretching may be inconsistent to that during walking^12^. Besides, it was criticized for its subjectivity, low test-retest and interrater reliability, as well as insufficient sensitivity to discriminate little changes after therapeutic interventions^18–21^. These shortcomings are shared with other resistance to passive movement (RTPM) tests, such as Modified Tardieu Scale (MTS) or the Tone Assessment Scale (TAS)^22,23^. Electrophysiological assessment, such as H-reflex and F-wave, can be useful to evaluate post-stroke spasticity^24^. However, the evoked responses do not discriminate clinical spasticity better than MAS^25^. Wearable sensor technologies have been applied to measurement of spasticity during walking, such as inertial measurement unit^26^, surface electromyography (sEMG) or myotonometry (or sonoelastography)^27^. For gait abnormality, it is not easy to distinguish the contribution of spasticity from that of muscle weakness, dystonia, synergy pattern or loss of sensation by using these sensors.

The pendulum or Wartenberg test is a standardized and quantifiable solution to evaluate knee spasticity. This gravity-provoked test was firstly introduced by Wartenberg in 1951^28^. The pendular movement of knee was observed by releasing a completely relaxed and maximally extended leg from horizon. The kinematic parameters, such as the angle of first swing excursion (FSE), resting angle (RA), angular velocity of FSE (ω(FSE)), and relaxation index (RI) could be extracted by electrogoniometry^9^, videography^29^, magnetic tracking^30^, even wearable Wii systems^31^. The pendulum test has excellent reliability and convergent validity^30,32,33^. The magnitudes of FSE and RI are significantly decreased in the paralytic limb after stroke^34^, and thus regarded as objective indexes inverse to knee spasticity. Nevertheless, the linkage between these parameters and mobility function was not fully investigated.

In this study, we hypothesized the pendular parameters may provide additional information to the MAS. There is no consensus regarding patient selection or outcome assessment for spasticity management. The pendulum test is a simple and reproducible assessment tool for knee spasticity. Although the pendulum test is historical, it has not yet been implemented in clinical routine practice. To the best of our knowledge, this is the first correlational study exploring connections between the pendulum parameters and mobility performance in chronic ambulant stroke population.

## Methods

### Study design

This cross-sectional study included patients with hemiplegic stroke and was approved by the Joint Institutional Review Board of Taipei Medical University (TMU-JIRB-201411053). All research was performed in accordance with relevant guidelines or regulations.

### Participants

The eligibility criteria were (1) age > 20; (2) stroke onset more than 6 months; (3) could walk independently with or without walking aid(s); (4) the diagnosis of stroke was confirmed by magnetic resonance imaging or computed tomography. The participants were excluded if they had (1) botulinum injection in the last 3 months (2) implantation of intrathecal baclofen pump; (3) instability, deformity or contracture (loss of more than one third of the maximal range of motion) of knees or ankles due to musculoskeletal disorders, autoimmune diseases or congenital diseases; (4) active arthritis or tendinitis with pain > 4 on a visual analog scale (0–10 total points) within 1 month; (5) prior surgical intervention of lower limbs; (6) language or cognitive impairment. Informed consent was obtained from all patients before recruitment. The following tests were performed consecutively in 1 day.

### Brünnstrom recovery stages

The Brünnstrom recovery stages define 6 stages of motor recovery after stroke^35,36^. A patient may plateau at any of these stages in the chronic phase (stroke onset more than 6 months). Stage I manifests flaccid of the affected limbs immediately following a stroke. Stage II presents developing spasticity, increased reflexes, and minimal synergic movement patterns. Stage III manifests prominent spasticity and voluntary control of the synergies but limited in range. Stage IV emerges isolated joint movements out of synergy and declining spasticity. Stage V manifests almost independence of synergies but limited in quality of movements. Stage VI indicates individual joint movements with minimal spasticity and is considered a full motor recovery.

### Tinetti test (TT)

The TT is a valid clinical test to assess an adult's static and dynamic balance. The risk of fall was scored on a scale of 28 points (12 for gait section and 16 for balance section)^37^. A score below 24 indicates significant risk of fall.

### 10-m walk test (10-MWT)

High intertester reliability of comfortable gait speed has been reported using 10-MWT^38^. With a 10-meter (3.28-feet) distance, marks were placed at 0, 2, 12 and 14 meters along a straight walkway. Participants started from the mark 0 and stopped at the mark 14. The timer was started when a leading foot crossing the mark 2, and was stopped when the foot crossing the mark 12. Each subject walked 3 rounds at a comfortable speed, and then another 3 rounds at the speed as fast as tolerated. The averages of both speeds in 3 rounds were documented.

### Timed up and go (TUG) test

The TUG test is a reliable assessment for dynamic stability and functional mobility^39^. It has been reported high correlations with Berg Balance scale, walking speed, stair-climbing test, and Barthel index^40,41^. In the study, the shortest time for a participant standing up from a back-support armchair, walking 3 meters, turning around, and sitting back to the chair was documented from 3 rounds.

### Pendulum test

**Figure 1:**
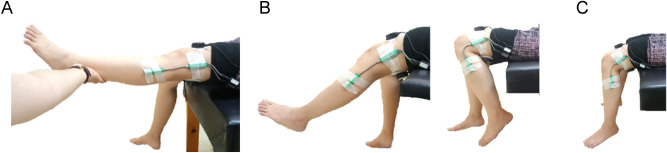
Setting of pendulum test. (**A**) Starting position: the leg was raised at the horizon of maximal knee extension with the subject in supine position. (**B**) Swing phase: after the leg totally relaxed, the heel is released and allowed to oscillate by the influence of gravity. (**C**) Resting position: when the oscillation stopped without visible movement, the difference of knee angle between the starting and resting position was measured as the resting angle (RA).

**Figure 2:**
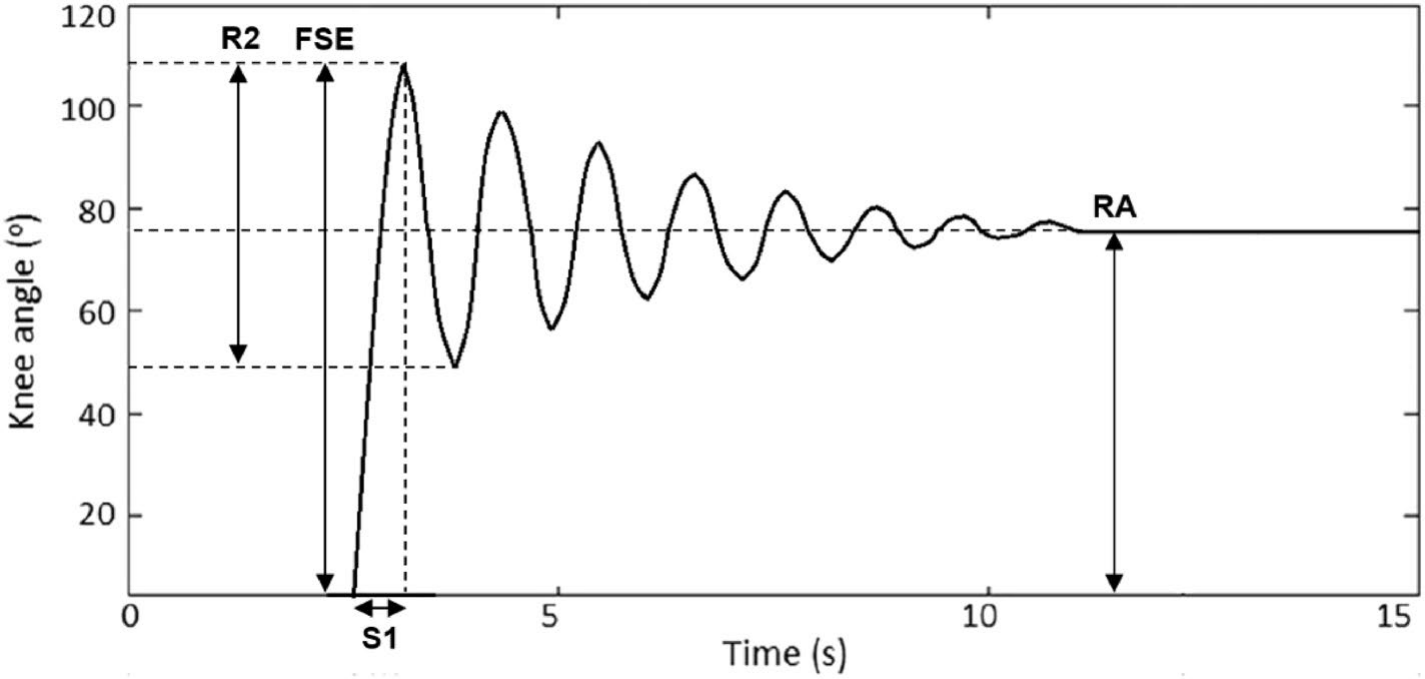
Oscillation of knee angle. A knee reaches the angle of first swing excursion (FSE) at time S1 and ended at the resting angle (RA) after several periods of oscillation. R2 is the angle of first extensive excursion. S1 is the duration of knee from the starting position to FSE.

The pendular parameters were extracted from an electrogoniometer with 2 sensors using ProComp Infiniti System (Thought of Technology Ltd., Quebec, Canada) and the digital raw data was filed by Biograph Infiniti software 5.1 (Thought of Technology Ltd., Quebec, Canada). The proximal sensor was attached to 15 cm above lateral femoral epicondyle, with its axis parallel to lateral femoral epicondyle and femoral gluteal tuberosity (Figure 1). The distal sensor was placed at 15 cm below lateral femoral epicondyle with its axis parallel to fibular head and lateral femoral epicondyle. Each subject began in the supine position with legs hanging freely off the edge of a mat table. The examiner held the tested leg until the subject was totally relaxed. Then the leg was released from the position of maximal knee extension and freely oscillated until no visible movement. The pendular parameters were defined as a previous study^42^ and is shown in Figure 2. The knee reached the angle of first swing excursion (FSE) at time S1 and ended at the resting angle (RA) after several periods of oscillation. The angular velocity (ω) of FSE is calculated by ω = FSE/S1. The relaxation index (RI) was defined as RI = FSE/RA. The test was repeated 10 times for both knees^43^.

### Modified Ashworth Scale (MAS)

The MAS test was performed by a female physiatrist with 10 years’ experience. The reliability of the MAS had been reported moderate to high (kappa were 0.54–0.84 and 0.59–0.83 for inter- and intra-rater comparison)^18,44^. Tonal abnormality was graded by 0 (no increase in muscle tone), 1 (slightly increase in muscle tone and giving a catch followed by minimal resistance at the end of range of motion(ROM)), 2 (slightly increase in muscle tone and giving a catch followed by minimal resistance throughout reminder of ROM), 3 (marked increase in muscle tone but the affected joint be easily moved), 4 (considerable increase in muscle tone, and difficult in passive movement) and 5 (rigid in flexion or extension)^18^. After a 5-minute rest, the patient was placed in a supine position. The extensor muscles of hip, knee and ankle segments were passively moved from the position of maximal extension to maximal flexion over 1 second. The flexor muscles of the 3 segments were moved from the position of maximal flexion to maximal extension over 1 second. The test was repeated 3 times for each muscle.

### Manual muscle testing (MMT) scale

The MMT is the most common assessment for muscle strength, which a muscle was tested against the examiner’s resistance and graded on 0 (no movement), 1 (visible movement), 2 (full active range of motion (aROM) with gravity counterbalanced), 3 (full aROM and against gravity), 4 (full aROM against some resistance), 5 (full aROM against strong resistance). After a 5-minute rest, the patient was placed in a supine position. Both extensor and flexor muscles of hips, knees and ankles were tested in the antigravity direction by the same physiatrist. Adequate stabilization was provided for unrelated joints to avoid unnecessary compensation. The examiner repeated the test 3 times for each muscle.

### Data analysis and statistical methods

The pendular kinematics and indexes were drawn and calculated using Matlab 2019a (The MathWorks, Inc., Massachusetts, U.S.A.). All variables were expressed as means and standard deviations. Spearmen and Pearson’s partial correlation tests were conducted between functional and spasticity parameters by using SPSS 22 (IBM, Armonk, NY, U.S.A.). The variables were age, sex, body mass index (BMI), Brünnstrom recovery stage, scores of total, balance and gait components of TT, the shortest time of TUG test, comfortable and the fastest speeds of 10-MWT, the MMT and the MAS of extensors and flexors of paretic hip, knee, and ankle segments. Age, sex and BMI have been reported correlated to function outcomes after stroke^45,46^. The significance level was set as *p* < 0.05. Multicollinearity was determined by the tolerance (1-R^2^) and variance inflation factor (VIF, 1/(1-R^2^)), where R^2^ is the coefficient of determination for the linear regression of a variable on all the other variables^47^. Tolerance less than 0.5 or VIF larger than 2 indicates the existence of multicollinearity. To identify the influence of individual spasticity rather than motor recovery on the mobility performance, Brünnstrom recovery stage was controlled in the partial correlation test.

## Results

### Demographic data

Forty participants (age 38–81, 67.5% males) were recruited from Taipei Medical University Hospital. Table 1 shows the demographic characteristics of the participants. 60% were ischemic stroke. The stroke lesions mostly located in basal ganglion (40%) and middle cerebral artery (37.5%). The disease duration ranged from 6 months to 20 years. The Brünnstrom recovery stage for paretic lower limb were III (n = 18), IV (n = 7) and V (n = 15). The mean Barthel index was 85.3 ± 14.9. All participants were able to walk without human assistance. Some participants needed either a walking aid (25%), ankle foot orthosis (15%) or both (24%). One patient had regularly taken muscle relaxant Tizanidine (1 mg three times a day).

**Table 1:** Demographic characteristics of the participants.

	Number (%)	Mean	SD
Age (years old)		58.9	11.6
**Sex**			
Male	27 (67.5%)		
Female	13 (32.5%)		
BMI (kg/m^2^)		25.1	4.5
Disease duration (months)		37.9	76.8
**Stroke type**			
Ischemic	24 (60%)		
Hemorrhagic	16 (40%)		
**Affected side (right/left)**			
Right	22 (55%)		
Left	18 (45%)		
**Lesion sites**			
Basal ganglion	16 (40%)		
Middle cerebral artery	15 (37.5%)		
Cerebellum	3 (7.5%)		
Anterior cerebral artery	2 (5%)		
Brain stem (Pons, Medulla)	2 (5%)		
B**runnstrom stage (lower limb)**		3.9	0.9
III	18 (45%)		
IV	7 (17.5%)		
V	15 (37.5%)		
Barthel index (scores)		85.3	14.9
**Assistive device**			
Walking aid	18 (45%)		
Ankle foot orthosis	14 (40%)		
Both	8 (24%)		

^§^*SD* standard deviation; *BMI* body mass index.

### Clinical assessment for mobility performance and spasticity

Table 2 shows the assessment of walking ability and spasticity. The average score of TT was 21.1 ± 4.4, with sectional scores of balance (12.2 ± 2.7) and gait (9.0 ± 2.6). The mean time of TUG test was 33.2 ± 20.0 seconds. The mean comfortable and the fastest speeds of 10-MWT were 0.46 ± 0.26 and 0.56 ± 0.33 m/s, respectively. The spasticity of paretic knee flexor and extensor on MAS were 0.08 ± 0.27 and 0.30 ± 0.56. The MMT and the MAS for paretic hip and ankle segments are shown in Supplementary table 1. As for the pendular parameters in paretic and non-paretic knees, FSE were 35.9 ± 22.9 and 45.9 ± 21.9 (*p* < 0.001), and RI were 1.3 ± 0.9 and1.62 ± 0.36 (*p* = 0.048), respectively.

**Table 2:** Clinical assessment, MAS, and the pendulum parameters.

	Mean	SD	Maximum	Minimum	Median (IQR)
**Tinetti (score)**	21.1	4.4	28	12	21.5 (6.25)
Balance section	12.2	2.7	16	6	13 (4)
Gait section	9.0	2.6	16	4	9 (4)
**10 MWT speed (m/s)**					
Comfortable	0.46	0.26	1.20	0.10	0.40 (0.30)
Fastest	0.56	0.33	1.30	0.10	0.48 (0.34)
TUG (second)	33.2	20.0	110.3	11.8	29.3 (19.4)
BI	85.3	14.9	100	65	87.5 (25)
**MMT**					
Knee extensor	3.75	0.81	5	0	4 (0)
Knee flexor	3.5	0.93	5	0	4 (1)
**MAS**					
Knee extensor	0.30	0.56	2	0	0 (0.25)
Knee flexor	0.08	0.27	1	0	0 (0)
**Pendulum test**					
Paretic knee					
FSE (degree)	35.9	22.9	91.1	3.6	33.0 (26.5)
RA (degree)	30.4	17.1	61.2	1.12	29.9 (28.3)
ω (FSE) (degree/s)	77.9	50.5	205.9	11.1	69.3 (50.7)
RI	1.30	0.90	6.54	0.48	1.20 (0.41)
Non-paretic knee					
FSE (degree)	45.9	21.9	105	6.14	39.9 (32.3)
RA (degree)	30.1	16.0	60.3	3.27	27.0 (25.9)
ω (FSE) (degree/s)	85.9	45.3	241.9	39.8	71.3 (53.0)
RI	1.62	0.36	2.65	1.17	1.58 (0.44)

^a^Data are presented as mean, standard deviation (SD), maximal, minimal, and interquartile range (IQR) values.

^§^*10 MWT* 10-m walk test; *TUG* timed-up-and-go test; *BI* Barthel index; *MMT* manual muscle test; *MAS* modified Ashworth scale; *FSE* first swing excursion; *ω(FSE)* angular velocity of the first swing excursion; *RA* resting angle of the pendulum test; *RI* relaxation index.

### Correlations between MMT, MAS, pendulum test and functional mobility

Table 3 shows the result of Spearman correlation test. The Brünnstrom stage correlated with all functional indexes, including the total, balance and gait scores of TT, comfortable and the fastest speeds of 10 MWT, time of TUG, as well as Barthel index (BI). The MMT of paretic knee extensor correlated with all function assessment except for the comfortable speed of 10-MWT. The MMT of hip flexor and extensor significantly correlated with the gait component and total score of TT (Supplementary table 1). The MMT of ankle extensor correlated with both gait component of TT, walking speeds, time of TUG and BI (Supplementary table 1). For paretic knee extensors, the MAS negatively correlated with the total (r = −0.313, *p* <0.01) and gait scores of TT (r = −0.433, *p* <0.01). The FSE positively correlated with the balance score of the TT (r =0.443, *p* <0.01). RI was separately correlated with both comfortable (r = 0.427, *p* <0.01) and the fastest speeds (r=0.327, *p* < 0.05) of 10-MWT. For each function scale, the tolerance of Brünnstrom stage, FSE, RI and MMT and MAS of knee extensor, were all larger than 0.5 with VIF < 2 in the collinearity test.

**Table 3:** Two-tailed Spearman correlation between MAS, pendulum parameters and mobility function.

	TT-Balance	TT-Gait	TT	10MWT-C	10MWT-F	TUG (s)	BI
Age	0.061 (.707)	0.049 (.763)	0.066 (.684)	− 0.020 (.903)	− 0.082 (.614)	0.042 (.795)	− 0.070 (.667)
Gender	− 0.039 (.813)	− 0.097 (.550)	− 0.081 (.620)	− 0.126 (.437)	− 0.083 (.610)	0.155 (.339)	− 0.157 (.333)
BMI	0.013 (.938)	− 0.109 (.502)	− 0.056 (.730)	0.077 (.636)	0.085 (.604)	− 0.237 (.141)	0.298 (.061)
Br. stage	0.364 (.021)*	0.409 (.009)**	0.463 (.003)**	0.460 (.003)**	0.505 (.001)**	− 0.382 (.015)*	0.386 (.014)*
MMT-KF	0.288 (.072)	0.313 (.049)*	0.333 (.036)*	0.101 (.535)	0.119 (.466)	− 0.195 (.227)	0.384 (.014)*
MMT-KE	0.353 (.025)*	0.400 (.011)*	0.437 (.005)**	0.293 (.067)	0.332 (.037)*	− 0.370 (.019)*	0.475 (.002)**
MAS-KF	0.228 (.157)	− 0.054 (.741)	0.095 (.560)	− 0.008 (.960)	− 0.021 (.900)	− 0.062 (.705)	− 0.042 (.798)
MAS-KE	0.009 (.957)	− 0.437 (.005)**	− 0.270 (.092)	− 0.157 (.335)	− 0.177 (.276)	0.139 (.392)	− 0.125 (.444)
FSE	0.443 (.004)**	0.061 (.707)	0.308 (.053)	− 0.139 (.393)	− 0.047 (.776)	0.003 (.984)	0.010 (.950)
ω (FSE)	0.295 (.064)	− 0.025 (.879)	0.167 (.303)	− 0.201 (.215)	− 0.137 (.399)	0.079 (.627)	− 0.150 (.355)
RA	0.417 (.007)**	− 0.002 (.991)	0.255 (.112)	− 0.186 (.249)	− 0.095 (.560)	0.021 (.895)	0.005 (.974)
RI	0.139 (.392)	0.256 (.111)	0.235 (.144)	0.427 (.006)**	0.327 (.039)*	− 0.162 (.318)	0.162 (.317)

^§^*BMI* body mass index; *Br. Stage* Brünnstrom recovery stage; *MMT* manual muscle test; *MAS* modified Ashworth scale; *KF* knee flexor; *KE* knee extensor; *FSE* first swing excursion; *ω(FSE)* angular velocity of first swing excursion; *RA* resting angle of the pendulum test; *RI* relaxation index; *TT* Tinetti test; *TT-Balance* balance section of TT; *TT-Gait* gait section of TT; *10 MWT* 10-m walk test; *10MWT-C* comfortable speed of 10 MWT; *10MWT-F* the fastest speed of 10 MWT; *TUG* timed-up-and-go test; *BI* Barthel index.

*Correlation is significant at the 0.05 level (2-tailed); **Correlation is significant at the 0.01 level (2-tailed).

^a^The MMT, MAS, and pendulum tests were investigated in the paretic knees (N = 40).

^b^The strength of the monotonic relationship is presented by the correlation coefficient *r*_*s*_. *p* values are given in parenthesis. Significant correlation are presented in bold.

### Partial correlations between MMT, MAS, pendulum test and functional mobility

Table 4 shows the result of partial correlation test. After controlling the factor of motor recovery (Brünnstrom stage), the MAS of paretic knee extensor stood negative correlation to the gait score of the TT (r = −0.355, *p* =0.027). The FSE positively correlated with the balance score of TT (r =0.318, *p* =0.018), but negatively correlation to the comfortable speed of 10-MWT (r = −0.322, *p* =0.046). The RI positively correlated with the comfortable speed of 10-MWT (r =0.367, *p* =0.022). The correlations between mobility performance and the MMT of hip and ankle segments were insignificant (Supplementary table 1).

**Table 4:** Two-tailed partial correlation controlling for Brünnstrom recovery stage.

	TT-Balance	TT-Gait	TT	10MWT-C	10MWT-F	TUG (s)	BI
MMT-KF	− 0.038 (.816)	− 0.062 (.707)	− 0.062 (.706)	− 0.133 (.420)	− 0.090 (.585)	0.212 (.194)	0.105 (.523)
MMT-KE	0.114 (.490)	0.038 (.818)	0.096 (.559)	− 0.048 (.774)	0.013 (.936)	− 0.038 (.816)	0.180 (.273)
MAS-KF	0.200 (.223)	− 0.137 (.407)	0.046 (.779)	− 0.034 (.838)	− 0.010 (.951)	− 0.036 (.830)	− 0.095 (.566)
MAS-KE	0.015 (.928)	− 0.355 (.027)*	− 0.205 (.212)	− 0.037 (.821)	− 0.037 (.824)	0.170 (.300)	− 0.051 (.757)
FSE	0.378 (.018)*	− 0.066 (.689)	0.204 (.212)	− 0.322 (.046)*	− 0.235 (.150)	0.130 (.430)	− 0.116 (.481)
ω (FSE)	0.268 (.099)	− 0.088 (.596)	0.120 (.467)	− 0.298 (.066)	− 0.239 (.143)	0.142 (.388)	− 0.221 (.177)
RA	0.284 (.080)	− 0.202 (.218)	0.062 (.709)	− 0.361 (.024)*	− 0.286 (.078)	0.152 (.356)	− 0.143 (.385)
RI	0.057 (.730)	0.178 (.278)	0.144 (.381)	0.367 (.022)*	0.246 (.131)	− 0.078 (.636)	0.078 (.639)

^§^*MMT* manual muscle test; *KF* knee flexor; *KE* knee extensor; *MAS* modified Ashworth Scale; *FSE* first swing excursion; *ω(FSE)* angular velocity of first swing excursion; *RA* resting angle of the pendulum test; *RI* relaxation index; *TT* Tinetti test; *TT-Balance* balance section of TT; *TT-Gait* Gait section of TT; *10 MWT* 10-m walk test; *10MWT-C* comfortable speed of 10 MWT; *10MWT-F* the fastest speed of 10 MWT; *TUG* timed-up-and-go test; *BI* Barthel index.

*Correlation is significant at 0.05 level.

^a^The MMT, MAS, and pendulum tests were investigated in the paretic knees (N = 40).

^b^The strength of the linear relationship is presented by the correlation coefficient *r*. *p* values are given in parenthesis.

## Discussion

The objectives of this study were to examine the correlations between the pendular parameters and mobility function, as well as to identify the role of knee spasticity in ambulant chronic stroke survivors. Supporting some of previous studies, knee spasticity significantly correlated with gait and balance function^4,11,12^. Our result shows the MAS of paretic knee extensor negatively correlated with the gait score of TT, while the FSE positively correlated with the balance section of TT. The RI correlated with the comfortable speed rather than the fastest speed of 10 MWT. These findings suggest a decrease of knee extensor spasticity indicates a better gait and balance, but a worse comfortable walking speed. The pendular parameters may provide additional information for functional evaluation, as complementary to the MAS.

The Brünnstrom recovery stage correlated with all functional indexes in the current study. Brünnstrom recovery stage reflects motor recovery and is an integral outcome of strength, selectivity and spasticity of the affected muscles^48^. Impairment of motor control was recognized as the most common cause of disability after stroke. Brünnstrom stage had been reported correlated with functional ambulation^48^, standing balance^49^ and functional fitness^50^. Motor recovery usually plateaus in chronic stroke patients^51^. After controlling the factor of motor recovery, the correlations between mobility and muscle strength (the MMT) of hips, knees and ankles became insignificant. Significant moderate correlation (|r| > 0.35) were still detected by pendular parameters FSE and RI. This finding suggests these parameters may contain some factors that affect chronic stroke mobility other than motor recovery.

Spasticity may cause impairment of gait in patients with subacute or chronic stroke^52^. Bohannon et al. concluded that in stroke patients, it was knee extensor torque rather than spasticity affected gait performance^8^. However, our data shows the MAS of paretic knee extensor was negatively correlated with the gait score of TT. This conflicting result may attribute to the participants’ phase of stroke. For the 17 stroke patients in the Bohannon’s study, the mean onset duration were 51 days, while in our study, most patients were at their chronic phase. For gait function, muscle strength is critical in the early phase of stroke, while spasticity may be more significant in chronic stroke.

Spasticity in stroke patients may also cause disturbance on balance^3^ and walking speed^53^. Both of which, however, were not correlated with the MAS in our result. Baetens et al. reported that the MAS of lower limb was not a risk factor of falling for stroke patients^54^. Bland et al. excluded admission MAS from a model predicting discharge 10-MWT speed in stroke inpatients^55^. We postulated that the MAS measured by the passive stretch test may be inconsistent to that during standing or walking^56^. Static spasticity can originate from hyperactive reflexes, and/or to stiffiness in muscles or connective tissues^57^. Conversely, dynamic spasticity may tend to increase coupling between muscle-tendon stretch velocity (or change of tissue length) and nonreflex muscular contraction^58^. Based on electromyography and kinetic measurements in hemiplegic stroke patients, the MAS of the paretic quadriceps had high correlations with the onset angle of resistance and the rate of change in resistance (or stiffness), but not with the peak resistance torque (or strength)^59^. The lack of dynamic characteristics possibly makes MAS difficult to discriminate the influence of spasticity on balance and walking speeds.

The pendulum test had been linked to assessment of spasticity due to its velocity-dependent nature, and it may further correlate to mobility function because of the dynamic and gravity-provoking features. Many studies assumed the swing phase of a normal human gait at preferred velocity is a passive movement and highly analogous to the swing of an unforced pendulum^60,61^. Spasticity can be reflexive (tonic or phasic) and nonreflexive (joint elastic stiffness or velocity-dependent resistances)^62^, which may be decomposable by the pendulum parameters. The FSE has been considered an inverse indicator of tonic hyperreflexia or overreactivity of quadriceps^63^, while the RA represents passive resistance (or stiffness) from both extensor and flexor, both of which compromise to the presence of an isotonic stretch (gravity)^64^. The RI, or FSE normalized by RA, was viewed as changes in length of muscle and soft tissue under resistance^64^, regarded as dynamic coupling consequence of muscle stiffness and the stretch (gravity). The decreases of FSE and RI in the paretic limbs after stroke had been confirmed^34,64,65^ and was also supported by our data.

The effect of knee spasticity on balance control after stroke is complex and not well understood^66^. To maintain standing balance, individuals with lower-limb spasticity may be difficult in moving the affected limbs^3^. Some stroke patients manifested hyperactive rectus femoris and biceps femoris during standing^67^, and the higher co-contraction level were associated with impaired balance and mobility^11,67^. Our data supports previous studies that spasticity has negative impact on standing balance, where positive correlation was remarked between the FSE and the balance score of TT. The FSE is the coupling consequence of gravity-provoked reflex and active resistance from both flexor and extensor. It may be capable of measuring co-contraction and as an indicator of balance function.

The relationship between spasticity and walking speed is debatable. Walking speed is a reliable and sensitive measure for functional status and overall health in a wide range of populations^68^. For stroke victims, walking speed is mainly affected by weakness in the affected hip flexors and knee extensors^8,69,70^. Ada et al. observed stroke patients exhibited tonic stretch reflexes during actions, but the EMG magnitude similar to that of the control subjects, suggesting spasticity may not cause problems in walking^10^. Soyuer et al. reported hypertonicity of leg extensor muscles may enable hemiparetic patients to support bodies during locomotion^4^. Our data demonstrated negative correlation between the FSE and comfortable speed, which means knee extensor spasticity may improve walking speed.

Our result also shows positive correlation between the RI and the comfortable speed of 10-MWT. Reduced muscle-tendon stretching velocity rather than hyperreflexia predominates the decrease of RI^65^. Muscular stiffness or low elasticity during active contraction may represent weakness of muscle, and further limits walking speed in patients with stroke. The correlation between RI and the comfortable speed has not been reported in other pendulum studies^8,10^. RI against spasticity was reported as a U-shape tendency with the bottom at the MAS 2^64^. Overreactivity of knee flexors may attribute to the reversal of the RI trend^64^, and usually coexists with spasticity of knee extensor. This co-contraction had been observed in subjects with severe spasticity, causing abnormal knee posture^11^ and inhibiting functional mobility^67^. In our study, most participants had mild to moderate (MAS: 0–2) knee extensor spasticity and mild (MAS: 0–1) knee flexor spasticity. The effect of spastic flexor was limited. This may explain the positive correlation between RI and the comfortable speed in our participants.

The pendulum test can be a reliable tool to evaluate mobility function in chronic stroke survivors. The pendular parameters, FSE and RI, can also be quantifiable indexes of spasticity influence on balance and walking speed. Dynamic coupling of knee flexor and extensor as well as elasticity of soft tissues may not be revealed by MAS. The constant gravity made the pendular parameters with high intra- and inter-rater reliability. Additional apparatus (such as electrogoniometry or videography) are required to extract pendulum parameters, which may somehow restrict the clinical application. Wearable technologies, such as Wii system, can be an alternative solution at low cost^31^. More clinical evidence is required for validity of these parameters, and extension to other spasticity-induced mobility impairment, such as cerebral palsy or stiff knee gait.

### Limitations

Several limitations need to be addressed. This study examined correlations between pendular parameters and mobility function only in a small sample size (N=40). Spasticity may diversely affect mobility in patients with various types of stroke. The pendulum test may not be applicable in cases with more severe spasticity^71^. In current study, the correlation coefficients were generally low. Larger sample size is required for validity of the pendular parameters. Second, the order of functional and manual tests may affect the results. The possible bias was originated from individual changes of spasticity after each test. Passive stretching, aerobic exercise, and anti-gravity standing have been recognized as physical treatments for spasticity^72^. Passive stretching may significantly reduce spasticity^72–74^. Aerobic exercise eliminates more spasticity than anti-gravity standing^72^. To minimize the bias, balance of TT was measured before gait, TUG and 10-MWT. Functional assessments were conducted before the Pendulum test and MAS. Even so, further study is required to clarify this ordering issue. Third, we chose MAS as the measurement tool for clinical spasticity because of its popularity and usability. Despite not an optimal scale, MAS is the most widely used clinical assessment for extremity spasticity^75^. However, there are several limitations of MAS. Some studies suggested stop using MAS due to questionable reliability and validity^44,76^, especially for ambiguity between the grade “1” and “1+”^77^. The modified modified Asworth scale (MMAS) and MTS may have higher sensitivity and reliability^22,77^, but not as popular as MAS^75^. In addition, using MAS can not distinguish dynamic shortening (exaggerated reflexes or clonus) from fixed shortening (stiffness or contracture) of a muscle^71^. Tardieu scale may be able to discriminate the difference^78^. Future research using MMAS or MTS for analyses should be considered. Fourth, all participants were tested by one experienced physiatrist in our study. We did not validate intra- or inter-rater reliabilities of MAS and the pendulum test since the reliabilities have been demonstrated moderate to high in the previous studies^18,30^. We did not control the stretching velocity during MAS test either. Future study involved preliminary reliability test should be concerned. Fifth, this cross-sectional study did not include time and patient factors into correlational analyses. The disease duration ranges from 6 months to 20 years. Time after stroke, individual physical and psychological conditions, comorbidities, development of compensatory skills for spasticity may greatly affect mobility and balance. We did not discuss these factors in this study. The last but not the least, this study cannot testify the existence of dynamic spasticity during gait. Sensor technologies, such as surface electromyography (sEMG), can be useful to detect dynamic spasticity. Nevertheless, international standard protocols, quantifiable parameters, and norm values are still missing in analyzing sEMG signals during walking^79^. Combining dynamic sEMG with the pendulum test can be useful to characterize the impact of spasticity on gait. Future studies are needed to establish a standard protocol for measurement of dynamic spasticity with reliability, validity, and discriminant ability.

## Conclusions

For patients with chronic stroke, the pendulum test can provide additional ambulatory information as complementary to MAS. After controlling the factor of motor recovery (Brünnstrom recovery stage) in partial correlation test, the MAS of paretic knee extensor negatively correlated with the gait component of TT while the FES of the pendulum test positively correlated to the balance section of TT. The comfortable speed of 10 MWT was associated with the FES and the RI. These results suggest a decrease of knee extensor spasticity may link to a better gait and balance, but a worst comfortable walking speed in chronic stroke victims. The pendulum test can be a potential tool for patient selection and outcome assessment after spasticity treatments in chronic stroke population.

## Supplementary Information

Supplementary Information.
