# Supplementary material for: Pendulum test in chronic hemiplegic stroke population: additional ambulatory information beyond spasticity
Source: Sci Rep. 2021 Jul 20;11:14769. doi: 10.1038/s41598-021-94108-5 (PMC8292373; doi:10.1038/s41598-021-94108-5)
Supplement: Supplementary file 1 — Supplementary Information. [file 41598_2021_94108_MOESM1_ESM.pdf]

Supplementary Information

	Mean	SD	Maximum	Minimum	Median(IQR)
MMT					
Hip flexor	3.85	0.43	5	3	4(0)
Hip extensor	3.75	0.42	5	2	4(0)
Ankle extensor	2.23	1.87	5	0	3(4)
Ankle flexor	2.43	1.96	5	0	4(4)
MAS					
Hip flexor	0.03	0.16	1	0	0(0)
Hip extensor	0.13	0.33	1	0	0(0)
Ankle extensor	0.03	0.15	1	0	0(0)
Ankle flexor	1.63	1.25	4	0	2(1.5)

Supplementary Table 1. Clinical assessment of hip and ankle segments. Data are presented as mean, standard deviation, maximal, minimal and median values. (Abbreviation: SD: standard deviation; MMT: manual muscle test; MAS: Modified Ashworth Scale; IQR: interquartile range)

	TT-Balance	TT-Gait	TT	10MWT-C	10MWT-F	TUG(s)	BI
MMT-HF	0.297(.063)	**0.380(.016)***	**0.432(.005)****	0.284(.076)	0.308(.053)	**-0.390(.013)***	**0.422(.007)****
MMT-HE	0.182(.260)	**0.379(.016)***	**0.351(.027)***	0.144(.375)	0.165(.308)	-0.233(.147)	**0.323(.042)***
MMT-AF	**0.315(.048)***	**0.401(.010)***	**0.376(.017)***	0.242(.133)	0.295(.065)	-0.268(.095)	0.265(.098)
MMT-AE	0.302(.058)	**0.464(.003)****	**0.396(.011)***	**0.353(.026)***	**0.392(.012)***	**-0.373(.018)***	**0.378(.016)***
MAS-HF	-0.098(.547)	-0.196(.226)	-0.167(.303)	-0.257(.110)	-0.201(.213)	0.257(.110)	-0.254(.114)
MAS-HE	-0.059(.715)	-0.201(.213)	-0.158(.331)	-0.134(.409)	-0.124(.444)	0.160(.323)	-0.117(.474)
MAS-AF	-0.229(.155)	-0.031(.851)	-0.083(.612)	-0.071(.661)	-0.082(.615)	0.128(.433)	-0.041(.802)
MAS-AE	-0.049(.764)	-0.245(.128)	-0.167(.303)	-0.194(.230)	-0.187(.247)	0.173(.285)	-0.233(.148)

*. Correlation is significant at the 0.05 level (2-tailed); **. Correlation is significant at the 0.01 level (2-tailed).

Supplementary table 2. Two-tailed Spearman correlation between clinical assessments of hip and ankle segments and mobility performance. The MMT and the MAS were investigated in the paretic knees (N=40). The strength of the monotonic relationship is given by the correlation coefficient r_s. P-values are given in parenthesis. Significant correlations are presented in bold. (Abbreviation: MMT: manual muscle test; MAS: Modified Ashworth Scale; HF: Hip flexor; HE: hip extensor; AF: ankle flexor; AE: ankle extensor; TT: Tinetti test; TT-Balance: Balance section of TT; TT-Gait: Gait section of TT; 10 MWT:10-Meter Walk Test; 10MWT-C: Comfortable speed of 10 MWT; 10MWT-F: the fastest speed of 10 MWT; TUG: timed-up-and-go test; BI: Barthel index)

	TT-Balance	TT-Gait	TT	10MWT-C	10MWT-F	TUG(s)	BI
MMT-HF	0.179(.276)	0.232(.155)	0.255(.116)	0.129(.433)	0.194(.236)	-0.093(.574)	**0.341(.034)***
MMT-HE	-0.066(.688)	0.091(.582)	0.012(.943)	-0.098(.551)	-0.082(.618)	-0.180(.274)	0.113(.494)
MMT-AF	-0.006(.969)	-0.012(.942)	-0.011(.945)	-0.175(.287)	-0.160(.332)	0.228(.163)	-0.199(.225)
MMT-AE	-0.056(.734)	0.062(.707)	0.001(.994)	-0.075(.649)	-0.062(.706)	0.166(.311)	-0.132(.425)

[a]. controlling for Brunnstrom stage *. Correlation is significant at 0.05 level.

Supplementary table 3.Two-tailed partial Pearson correlation between muscle strength of hip and ankle segments and ambulatory function controlling for Brünnstrom stage. The MMT tests were investigated in the paretic knees (N=40). The strength of the linear relationship is given by the correlation coefficient r. P-values are given in parenthesis. Significant correlations are presented in bold. (Abbreviation: MMT: manual muscle test; HF: Hip flexor; HE: hip extensor; AF: ankle flexor; AE: ankle extensor; TT: Tinetti test; TT-Balance: Balance section of TT; TT-Gait: Gait section of TT; 10 MWT:10-Meter Walk Test; 10MWT-C: Comfortable speed of 10 MWT; 10MWT-F: the fastest speed of 10 MWT; TUG: timed-up-and-go test; BI: Barthel index)
